# Prognostic value of coronary microvascular dysfunction assessed by coronary angiography-derived index of microcirculatory resistance in diabetic patients with chronic coronary syndrome

**DOI:** 10.1186/s12933-022-01653-y

**Published:** 2022-10-29

**Authors:** Wen Zhang, Shekhar Singh, Lu Liu, Abdul-Quddus Mohammed, Guoqing Yin, Siling Xu, Xian Lv, Tingting Shi, Cailin Feng, Rong Jiang, Ayman A. Mohammed, Redhwan M. Mareai, Yawei Xu, Xuejing Yu, Fuad A. Abdu, Wenliang Che

**Affiliations:** 1 Department of Cardiology, Shanghai Tenth People’s Hospital, Tongji University School of Medicine, 301 Yanchang Road, 200072 Shanghai, China; 2 Department of Cardiology, Shanghai Tenth People’s Hospital, Chongming branch, Shanghai, China

**Keywords:** Chronic coronary syndrome, Diabetes mellitus, Coronary microvascular dysfunction, Coronary angiography‑derived index of microvascular resistance, Outcome

## Abstract

**Background:**

Coronary microvascular dysfunction (CMD) is common and is associated with unfavorable cardiovascular events in patients with diabetes mellitus (DM). Coronary angiography-derived index of microcirculatory resistance (caIMR) is a recently developed wire- and hyperemic agent-free method to assess CMD. We aimed to investigate the prognostic impact of CMD assessed by caIMR on clinical outcomes in patients with DM and chronic coronary syndrome (CCS).

**Methods:**

CCS patients who underwent coronary angiography between June 2015 to May 2018 were included. Coronary microvascular function was measured by caIMR, and CMD was defined as caIMR ≥ 25U. The primary endpoint was major adverse cardiac events (MACE). Kaplan-Meier analysis and Cox proportional hazards models were used to assess the relationship between caIMR and the risk of MACE.

**Results:**

Of 290 CCS patients, 102 patients had DM. Compared with non-diabetic patients, CMD (caIMR ≥ 25U) was higher among DM patients (57.8% vs. 38.3%; p = 0.001). During a mean 35 months follow-up, 40 MACE had occurred. Patients with caIMR ≥ 25 had a higher rate of MACE than patients with caIMR < 25 (20.6% vs. 8.2%, p = 0.002). Of these, the MACE rate was higher among DM patients with caIMR ≥ 25 than those with caIMR < 25 (33.9% vs. 14.0%; p = 0.022). In multivariable Cox analysis, caIMR ≥ 25 was independently associated with MACE in the DM patients but not in non-DM patients (HR, 2.760; 95% CI, 1.066–7.146; P = 0.036).

**Conclusion:**

CMD assessed by caIMR was common and is an independent predictor of MACE among diabetic patients with CCS. This finding potentially enables a triage of higher-risk patients to more intensive therapy.

**Supplementary Information:**

The online version contains supplementary material available at 10.1186/s12933-022-01653-y.

## Background

Diabetes mellitus (DM) is a common risk factor for ischemic heart disease, and its prevalence is increasing globally [1, 2]. There is clear evidence that DM is associated with adverse cardiovascular risk and mortality in patients with the chronic coronary syndrome (CCS) [3–6]. Coronary microvascular dysfunction (CMD) is a clinical condition where the structure and/or function of coronary microvessels is affected [7–10]. It is increasingly recognized that CMD is involved in the pathogenesis of multiple cardiovascular diseases; in DM patients, CMD is characterized by impaired vasodilation in response to increased oxygen demand, which may occur as early manifestations of DM [1, 10, 11]. Poor glycemic management has reportedly been linked to CMD in diabetic individuals with chest pain and non-obstructive coronary artery disease (CAD) [12]. Observational studies have shown that CMD is associated with excess risk and can be prognostically useful to predict cardiac death in DM patients regardless of traditional cardiovascular risk factors [13–18]. Therefore, timely recognition of CMD in DM patients is crucial in preventing adverse outcomes and improving their quality of life.

Several invasive and non-invasive approaches for assessing CMD have been established [19–23]. Among these, the index of microcirculatory resistance (IMR) has been used increasingly, which is based on the thermodilution method measured by a pressure-temperature sensor guidewire [24–26]. Previous studies have shown a significant association of IMR with clinical outcomes among patients with ST-elevated myocardial infarction (STEMI), non–ST-segment elevation myocardial infarction (NSTEMI), and stable CAD [27–30]. However, its application within routine clinical practice remains extremely limited because of its invasive nature, longer procedural time, increased cost, technical complexity mainly related to pressure wire manipulation, and the use of adenosine to induce maximal hyperemia. Alternatively, advances in interventional cardiology enable the development of a novel physiological index of microvascular resistance derived from coronary angiography (caIMR), which eliminates the need for a pressure wire and hyperemic adenosine has been recently introduced to overcome these issues [31–34]. Robust studies evaluating the role of caIMR and clinical outcomes reported that caIMR is a strong predictor of risk in patients with STEMI [35] and myocardial infarction with non-obstructive coronary arteries (MINOCA) [36]. However, the prognostic implication of caIMR among DM patients with CCS has not been evaluated.

Therefore, this study aimed to evaluate the prognostic impact of CMD assessed by non-invasive caIMR in DM patients presenting with CCS and to elucidate whether caIMR can provide any potential clinical significance in this patient population.

## Materials and methods

### Study population

This is a retrospective observational study that enrolled CCS patients who underwent coronary angiography for suspected angina (based on clinical symptoms and/or signs of ischemia, ECG findings, and clinical risk profiles) at Shanghai Tenth People’s Hospital between June 2015 - May 2018. Patients who were diagnosed with CCS according to the 2019 ESC guidelines for the diagnosis and management of CCS [3] and age > 18 years old were included in the present study. The exclusion criteria include: (1) myocardial infarction (MI) within seven days; (2) severe hepatic or renal disorders; (3) any type of malignant tumor; (4) left ventricular ejection fraction < 35%; and (5) post coronary artery bypass graft surgery (CABG). The caIMR exclusion criteria were based on a previous study [31] which include: (1) low contrast opacification; (2) apparent vascular overlap or distortion of the target vessel; and (3) the poor angiographic image quality unable to provide a contour detection requested by the FLASH software.

Demographic and baseline clinical information data of all participants were recorded from medical files. Fasting blood samples were obtained after admission to measure fasting blood glucose (FBG), hemoglobin A1c (HbA1c), total cholesterol (TC), creatinine, and low-density lipoprotein (LDL). The detailed data on echocardiography and coronary angiography were collected from examination report sheets.

Our study was carried out in accordance with the Helsinki Declaration and was approved by the ethical review board of Shanghai Tenth People’s Hospital. Each participating patient in this study recruited written informed consent.

### caIMR measurement

caIMR is measured by two trained cardiologists using the software with the FlashAngio system (FlashAngio, Rainmed Ltd., Suzhou, China), who were blinded to the patients’ baseline information and outcomes. The detailed theory for caIMR measurement has been described by a previous study [31], which is calculated as the following equation:

$${\text{caIMR}}={\left( {{\text{Pd}}} \right)_{{\text{hyp}}}}\frac{{\text{L}}}{{{\text{K}} \cdot {{\text{V}}_{{\text{diastole}}}}}}$$

In brief, caIMR was analyzed by performing three steps; (1) 3D network of mesh was generated along the epicardial artery. (2) The hyperemic aortic pressure (Pd)_hyp_ was calculated based on two mean aortic pressure values. (3) The caIMR was computed using the above equation. In the above equation, (Pd)_hyp_ is the mean pressure (unit: mmHg) at the distal position at the maximal hyperemia, K is a constant (K = 2.1) calculated from a previous study, L is a constant (non-dimensional) that mimics the length from the inlet to the distal position (L = 75, mimicking 75 mm downstream from the inlet of coronary arterial tree), and V_diastole_ is the mean flow velocity (unit: mm/s) at the distal position at diastole and V_hyp_ = K · V_diastole_, refers to the mean flow velocity (unit: mm/s) at the distal position at the maximal hyperemia.

A total of 290 patients were measured for caIMR. The caIMR was measured in 322 stenotic epicardial arteries. Those who underwent percutaneous coronary intervention (PCI) had their caIMR measured after the PCI procedure. If a patient had multiple coronary stenosis lesions, the highest caIMR value was used. caIMR measurement was accomplished by 2 experienced cardiologists without any awareness of experiment outcomes.

### Definitions and cut-off values

Diabetes was defined as following: (1) HbA1c ≥ 6.5 %; (2) Random plasma glucose ≥ 200 mg/dl (≥ 11.1 mmol/l); (3) FBG ≥ 126 mg/dl (≥ 7.0 mmol/l); and (4) OGTT 2‑hour glucose in venous plasma ≥ 200 mg/dl (≥ 11.1 mmol/l) [37]. The diabetes patients in this study were all type 2 diabetes.

Hyperlipidemia is defined by total cholesterol, triglyceride, or LDL level higher than the 90th percentile or an HDL level lower than the 10th percentile for the general population. Hypertension is defined as BP levels in the range of ≥ 140 mmHg systolic or ≥ 90 mmHg diastolic. CMD was defined as caIMR ≥ 25U, according to the established cut-off value [31].

### Follow up and endpoint of the study

All patients were followed up for 35 months by their physician at Shanghai Tenth People’s Hospital through phone calls and outpatient visits. The primary endpoint of our study was major adverse cardiac events (MACE), including cardiovascular death, nonfatal myocardial infarction (MI), heart failure, and ischemia-driven revascularization. Cardiovascular death refers to death due to acute MI, sudden cardiac death, heart failure, stroke, cardiovascular procedures, cardiovascular hemorrhage, and other cardiovascular causes. Nonfatal MI was defined as symptoms of myocardial ischemia with the dynamic changes in cardiac biomarkers [38]. Heart failure is diagnosed according to the ESC Guidelines for diagnosing and treating acute and chronic heart failure [39]. Ischemia-driven revascularization refers to the revascularization procedure due to recurrent angina and/or positive test for ischemia.

### Statistical analysis

The present study data were analyzed with the Statistical Package for Social Sciences (SPSS) v.22. Figures were constructed by GraphPad softwarev.8.0.1. Numerical variables were expressed as the mean ± standard deviation with a normal distribution, and categorical variables were displayed as percentages. The independent sample t-test is used for intergroup comparisons of numerical variables. The chi-square and Fisher’s exact tests were used to compare categorical variables. Kaplan-Meier analysis was used to calculate the MACE-free survival rates, and differences were evaluated using the log-rank test. The association between caIMR and the outcomes was determined using Cox proportional regression analysis. Univariate analyses were performed to assess the association between each variable (listed in Table 1) along with caIMR and the clinical outcome. Univariate predictors with P < 0.10 were variables in covariates for multivariable models. The hazard ratio (HR) with a 95% confidence interval (CI) was estimated. The assumption of proportional hazard was tested by a visual examination of the log (minus log) curves. All analysis was conducted two-sided, and statistical significance was identified at P-value < 0.05.

**Table 1:** Baseline characteristics of the study population

	All (n = 290)	DM (n = 102)	Non-DM (n = 188)	P-value
**General characteristics**				
Age (years)	64.87 ± 9.21	66.29 ± 8.49	64.24 ± 9.72	0.074
Male, n (%)	201 (69.3)	68 (66.7)	133 (70.7)	0.472
**Cardiovascular risk factors**				
BMI (kg/m2)	25.00 ± 3.07	25.59 ± 3.21	24.67 ± 2.87	0.014
Smoking history, n (%)	68 (23.4)	22 (21.6)	46 (24.5)	0.578
Hypertension, n (%)	203 (70.0)	75 (73.5)	128 (68.1)	0.334
Hyperlipidaemia, n (%)	60 (20.7)	17 (16.7)	43 (22.9)	0.213
Atrial fibrillation, n (%)	15 (5.2)	6 (5.9)	9 (4.8)	0.688
PCI performed, n (%)	229 (79.0)	86 (84.3)	143 (76.1)	0.100
LVEF (%)	62.28 ± 4.84	59.48 ± 8.69	62.04 ± 5.64	0.010
CKD, n (%)	22 (7.6)	14 (13.7)	8 (4.3)	0.004
CAD, n (%)	276 (95.2)	99 (97.1)	177 (94.1)	0.414
1- vessel disease	115 (39.7)	40 (39.2)	75 (39.9)	0.910
2- vessel disease	100 (34.5)	34 (33.3)	66 (35.1)	0.762
3- vessel disease	61 (21.0)	25 (24.5)	36 (19.1)	0.285
**Laboratory findings**				
FBG (mmol/L)	5.90 ± 2.01	6.98 ± 2.59	5.30 ± 1.02	< 0.001
HbA1c (%)	6.49 ± 1.27	7.40 ± 1.38	6.00 ± 0.77	< 0.001
TC (mmol/L)	3.65 ± 0.98	3.61 ± 0.98	3.66 ± 0.95	0.691
LDL-C (mmol/L)	1.95 ± 0.87	1.91 ± 0.81	1.97 ± 0.87	0.581
Cr (umol/L)	77.40 ± 20.26	78.04 ± 22.89	77.05 ± 18.72	0.709
eGFR (mL/min/1.73m^2^)	90.76 ± 21.51	90.40 ± 23.66	90.96 ± 20.30	0.833
**Cardiovascular medical therapy**				
Aspirin, n (%)	260 (89.7)	94 (92.2)	166 (88.3)	0.303
P2Y12 receptor antagonist, n (%)	233 (80.3)	85 (83.3)	148 (78.7)	0.346
Statin, n (%)	278 (95.9)	95 (93.1)	183 (97.3)	0.159
ACEI/ARB, n (%)	159 (54.8)	62 (60.8)	97 (51.6)	0.133
Beta blocker, n (%)	168 (57.9)	60 (58.8)	108 (57.4)	0.821
CCB, n (%)	149 (51.4)	45 (44.1)	104 (55.3)	0.068

*DM* diabetes mellitus, *BMI* body mass index, *PCI* percutaneous coronary intervention, *CKD* chronic kidney disease, *CAD* coronary artery disease, *LVEF* left ventricular ejection fraction, *FBG* fasting blood glucose, *HbA1c* Hemoglobin A1c, *TC* total cholesterol, *LDL-C* low-density lipoprotein-cholesterol, *Cr* creatinine, *eGFR* estimate glomerular filtration rate, *ACEI/ARB* angiotensin-converting-enzyme inhibitors/angiotensin receptor blockers, *CCB* calcium channel blocker

## Results

### Baseline characteristics

**Fig. 1:**
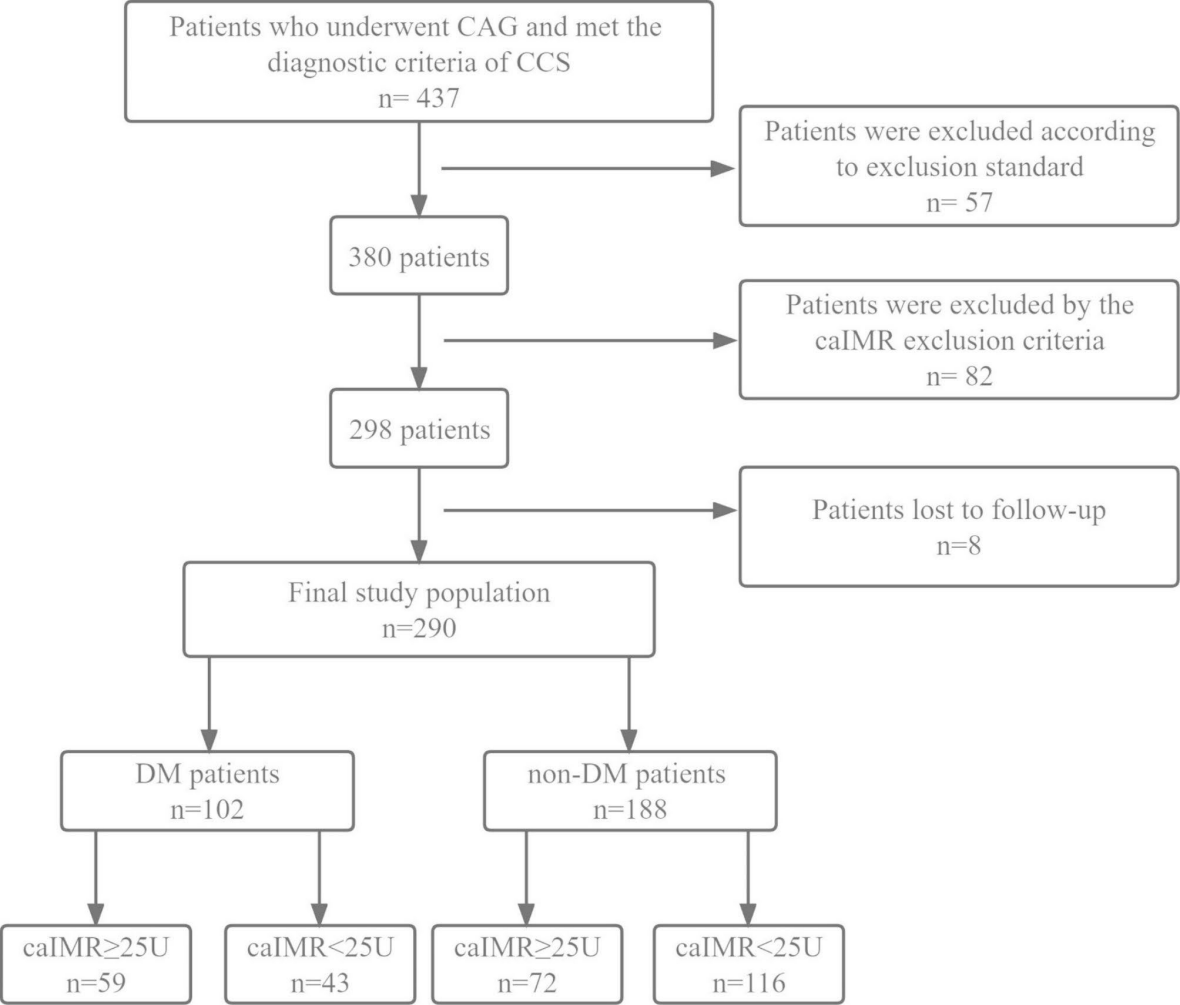
Flowchart of the study selection process. CAG, coronary angiography; CCS, chronic coronary syndrome; caIMR, coronary angiography-derived index of microcirculatory resistance; DM, diabetes mellitus

A total of 437 patients who underwent CAG and met the diagnostic criteria of CCS were included in this study, in which 57 patients were excluded according to the exclusion standard, 82 patients were excluded due to the caIMR exclusion criteria, and eight patients were lost to follow up. Two hundred and ninety patients were finally included in the analysis of this study, in which 229 (79.0%) underwent PCI procedure. Among these, 102 (35.2%) patients had DM, while 188 (64.8%) were without DM, and the CMD (caIMR ≥ 25U) was found in 131 (45.2%) patients among the total CCS population (Fig. 1). The median duration of diabetes was 6 (3–10) years.

**Fig. 2:**
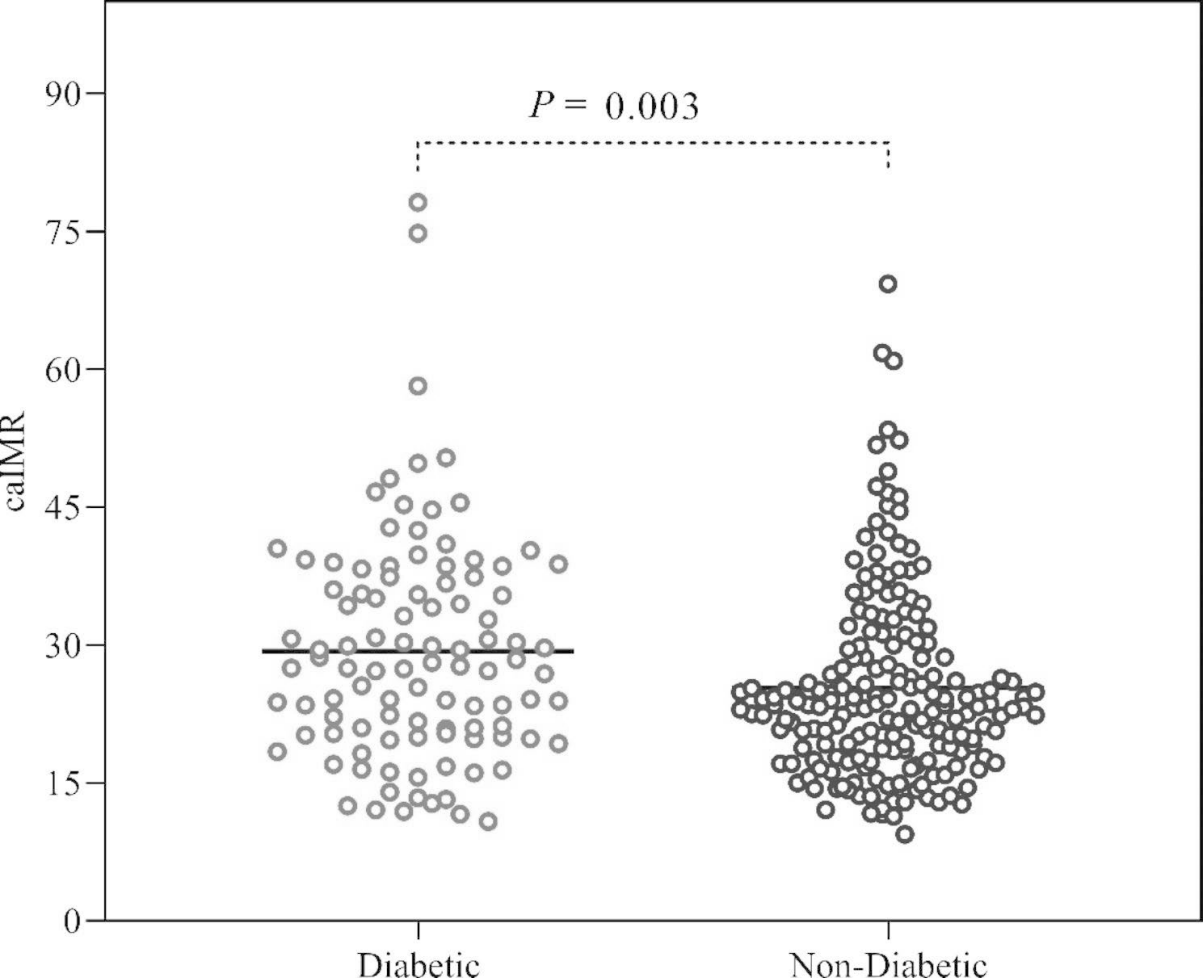
Scatter plot of caIMR between DM patients and those without DM. caIMR: coronary angiography‑derived index of microcirculatory resistance

Baseline characteristics, laboratory findings, and cardiovascular medications of the study population are shown in Table 1. Body mass index, FBG, HbA1c levels, and the rate of chronic kidney disease were higher in the DM patients compared to the non-diabetic patients, whereas LVEF was lower in DM patients. The distribution of other baseline characteristics and laboratory information data showed non-significant differences between the two groups. There was also no significant difference in the prevalence of CAD and the number of vessels disease between caIMR above and below 25 (Additional file 1: Table S1). In addition, the two groups detected no significant differences in the use of cardiovascular medications. The glucose-lowering medications for the diabetic cohort are displayed in Additional file 1: Table S2. The Table 2 displays the coronary microvascular function among the study population. Coronary microvascular function assessed by caIMR was measured in 322 target coronary arteries: right coronary artery (RCA), n = 71 (22.0%); left anterior descending artery (LAD), n = 203 (63.0%); and left circumflex artery (LCX), n = 48 (14.9%). The prevalence of CMD (caIMR ≥ 25U) was higher among DM patients compared with non-diabetic patients (57.8% vs. 38.3%; p = 0.001). Furthermore, a significant difference was observed in the caIMR value between the 2 groups. DM patients had a significantly higher caIMR value than those without DM (29.35 ± 12.25 vs. 25.36 ± 10.30 P = 0.003) (Table 2; Fig. 2).

**Table 2:** Physiological parameters of target vessels in the study population

	All (n = 290)	DM (n = 102)	Non-DM (n = 188)	P-value
**Coronary physiological parameters**				
caFFR	0.92 ± 0.06	0.92 ± 0.06	0.91 ± 0.06	0.194
caIMR	27.34 ± 10.79	29.35 ± 12.25	25.36 ± 10.30	0.003
caIMR ≥ 25	131 (45.2)	59 (57.8)	72 (38.3)	0.001
**Target vessel**	322	115	207	
LAD, n (%)	203 (63.0)	71 (61.7)	132 (63.8)	0.718
LCX, n (%)	48 (14.9)	17 (14.8)	31 (15.0)	0.963
RCA, n (%)	71 (22.0)	27 (23.5)	44 (21.3)	0.645

*DM* diabetes mellitus, *caFFR* coronary angiography-derived fractional flow reserve, *caIMR* coronary angiography-derived index of microcirculatory resistance, *LAD* left anterior descending branch, *LCX* left circumflex coronary artery, *RCA* right coronary artery

### Clinical outcome

**Fig. 3:**
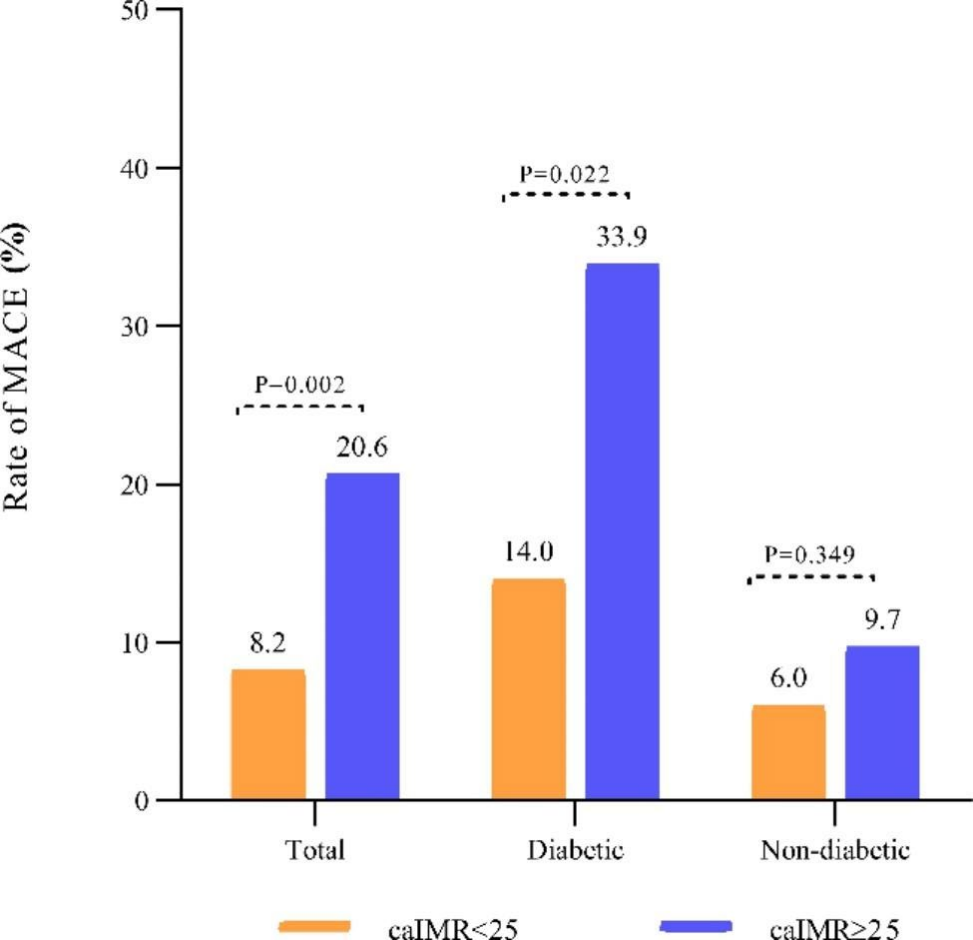
Rate of MACE among total, DM and non-DM patients according to caIMR. caIMR, coronary angiography-derived index of microcirculatory resistance; MACE, major adverse cardiac events

**Fig. 4:**
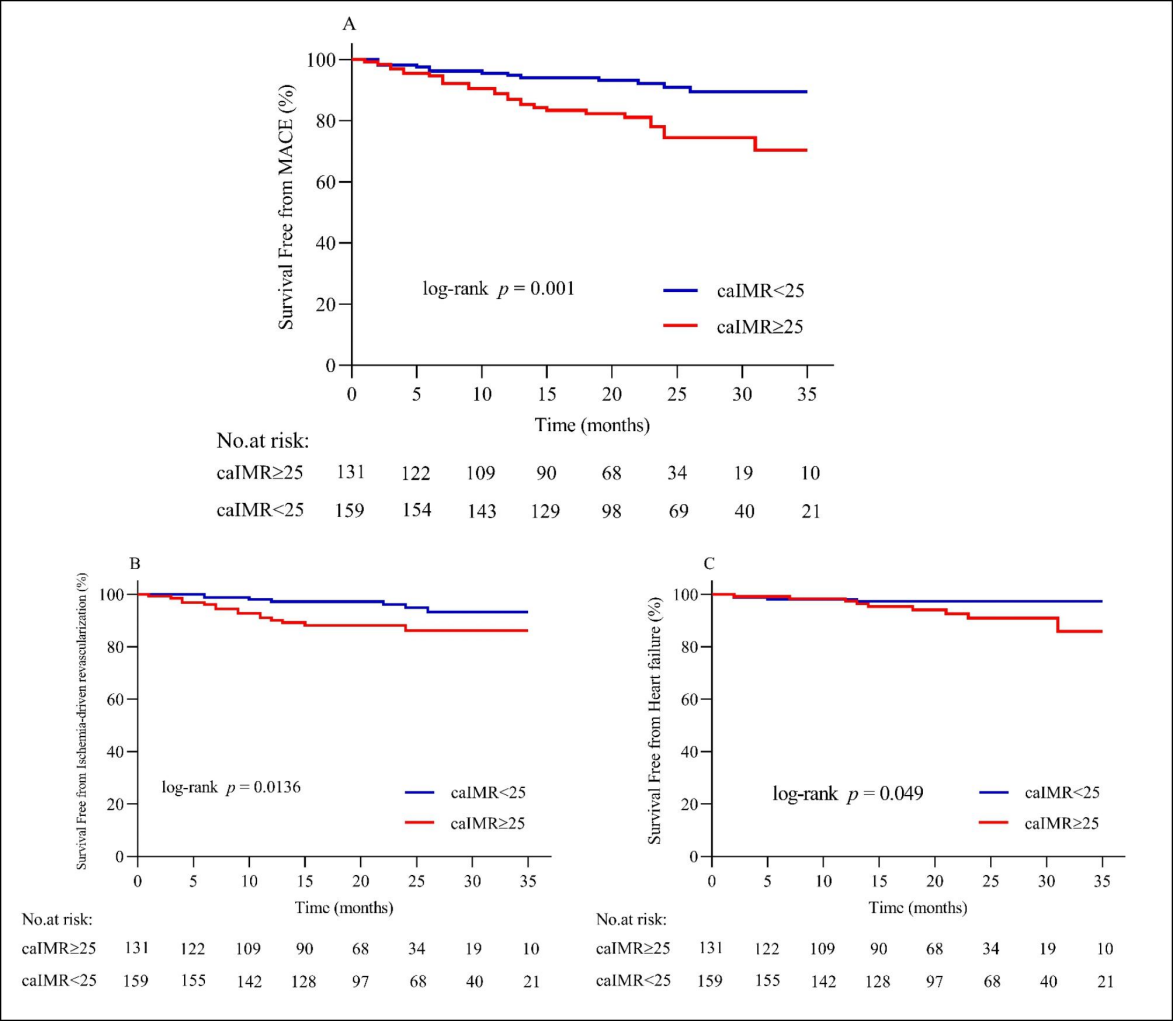
(**A**) Kaplan-Meier survival curves of MACE in CCS patients according to caIMR; (**B**) Kaplan-Meier survival curves of ischemia-driven revascularization in CCS patients according to caIMR; (**C**) Kaplan-Meier survival curves of heart failure in CCS patients according to caIMR. caIMR, coronary angiography-derived index of microcirculatory resistance

**Fig. 5:**
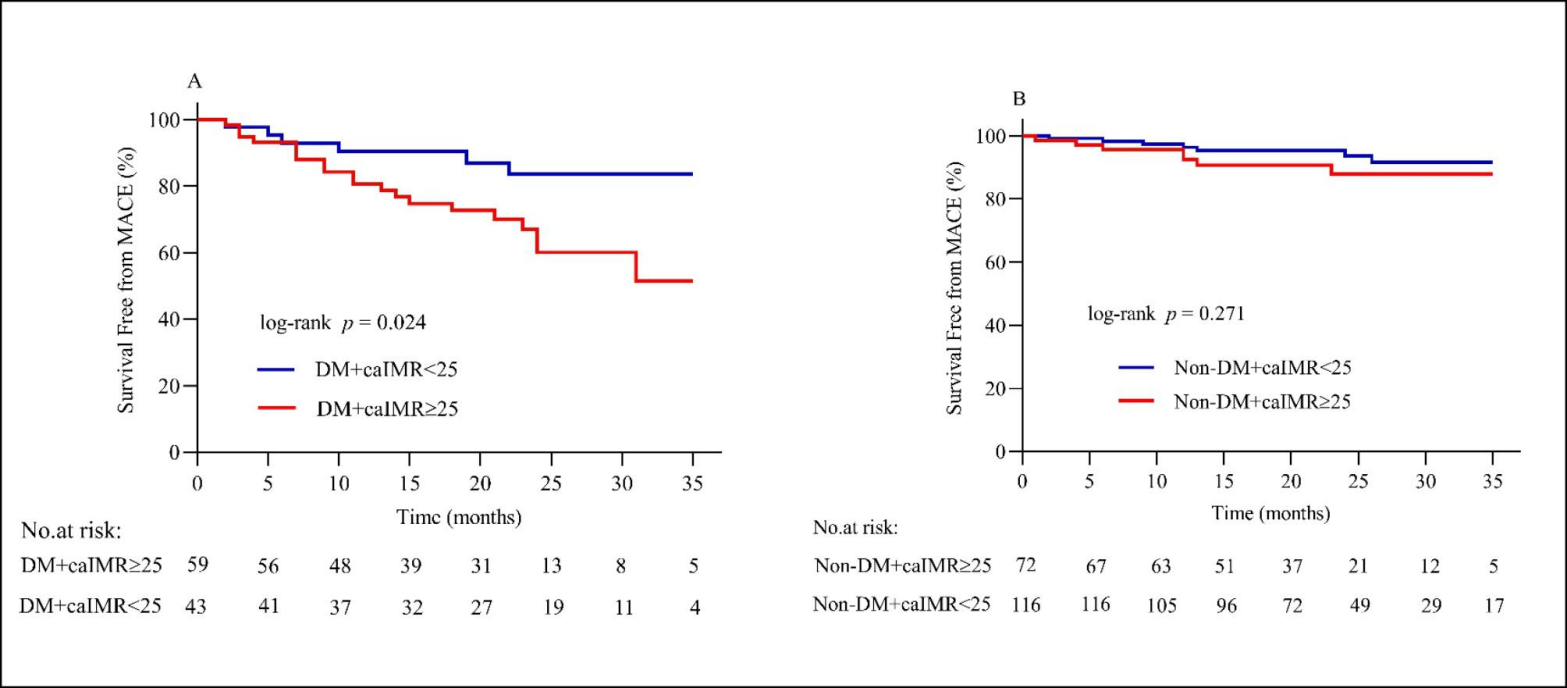
(**A**) Kaplan-Meier survival curves of MACE in DM patients with CCS according to caIMR; (**B**) Kaplan-Meier survival curves of MACE in non-DM patients with CCS according to caIMR. DM, diabetes mellitus; caIMR, coronary angiography-derived index of microcirculatory resistance

The mean follow-up duration was 35 months. Forty MACE were recorded during the follow-up duration among the total CCS population. Patients with CMD (caIMR ≥ 25U) had a notably higher rate of MACE as compared to non CMD patients (caIMR < 25) (20.6% vs. 8.2%, p = 0.002) (Table 3; Fig. 3). Kaplan-Meier survival curves also demonstrated a significantly high MACE in patients with caIMR ≥ 25 than in caIMR < 25 patients (log-rank P = 0.001) (Fig. 4.A). Similarly, the same results were observed in patients with caIMR ≥ 25 when ischemia-driven revascularization or heart failure was analyzed separately (log-rank P = 0.0136, and 0.049, respectively) (Fig. 4. B.C). The incidence rate of MACE was higher in DM patients with caIMR ≥ 25 than in the caIMR < 25 group (33.9% vs. 14.0%; p = 0.022). In contrast, the incidence rate of MACE was not significantly different between caIMR ≥ 25 and caIMR < 25 in non-DM patients (9.7% vs. 6.0%; p = 0.349) (Table 3; Fig. 3). Kaplan-Meier curves analysis also showed a significantly increased risk of MACE in DM patients with caIMR ≥ 25 (log-rank P = 0.024) (Fig. 5. A). In contrast, there is no difference in non-diabetic patients between the high and low caIMR groups (log-rank P = 0.271) (Fig. 5. B).

**Table 3:** Patients outcomes

	ALL		P-value	DM		P-value	Non-DM		P-value
	caIMR ≥ 25 (n = 131)	caIMR < 25 (n = 159)		caIMR ≥ 25 (n = 59)	caIMR < 25 (n = 43)		caIMR ≥ 25 (n = 72)	caIMR < 25 (n = 116)	
**MACE**	27 (20.6)	13 (8.2)	0.002	20 (33.9)	6 (14.0)	0.022	7 (9.7)	7 (6.0)	0.349
Cardiovascular death	0	1 (0.6)	1.000	0	0		0	1 (0.9)	1.000
Nonfatal MI	3 (2.3)	1 (0.6)	0.483	2 (3.4)	1 (2.3)	1.000	1 (1.4)	0	0.383
Heart failure	9 (6.9)	4 (2.5)	0.075	8 (13.6)	2 (4.7)	0.247	1 (1.4)	2 (1.7)	1.000
Ischemia-driven revascularization	15 (11.5)	7 (4.4)	0.024	10 (16.9)	3 (7.0)	0.136	5 (6.9)	4 (3.4)	0.459

*DM* diabetes mellitus, *MACE* major adverse cardiovascular events, *MI* myocardial infarction, *caIMR* coronary angiography-derived index of microcirculatory resistance

### Association between caIMR and clinical outcomes

The association between caIMR and clinical outcomes is shown in Tables 4 and 5. Univariate Cox analysis showed that caIMR ≥ 25 was a significant independent predictor associated with increased risk of MACE among the total CCS population (HR, 2.857; 95% CI, 1.472–5.542; P = 0.002) (Table 4). caIMR ≥ 25 was only independently associated with MACE in the DM patients but not in the non-DM patients (HR, 2.731; 95% CI, 1.095–6.814; P = 0.031 vs. HR, 1.786; 95% CI, 0.626–5.099; P = 0.278, respectively). Multivariable Cox analysis showed that caIMR ≥ 25 remained strongly correlated with the risk of MACE in DM patients even after adjusting for additional confounding risk factors (HR, 2.760; 95% CI, 1.066–7.146; P = 0.036) (Table 5).

**Table 4:** Univariate analysis for clinical outcome

	All		DM		Non-DM	
	HR (95% CI)	P-value	HR (95% CI)	P-value	HR (95% CI)	P-value
caIMR ≥ 25	2.857 (1.472–5.542)	0.002	2.731 (1.095–6.814)	0.031	1.786 (0.626–5.099)	0.278
Male	1.955 (0.901–4.245)	0.090	2.469 (0.929–6.563)	0.070	1.605 (0.448–5.755)	0.468
BMI	1.045 (0.949–1.150)	0.375	0.961 (0.853–1.083)	0.514	1.101 (0.925–1.310)	0.279
Hyperlipidemia	0.594 (0.232–1.520)	0.277	0.387 (0.091–1.641)	0.198	1.067 (0.295–3.856)	0.922
Hypertension	0.734 (0.387–1.392)	0.343	0.473 (0.217–1.032)	0.060	1.245 (0.390–3.971)	0.711
LVEF	0.907 (0.878–0.937)	< 0.001	0.929 (0.895–0.965)	< 0.001	0.888 (0.830 − 0.951)	0.001
Age	1.012 (0.979–1.046)	0.488	0.995 (0.951–1.041)	0.820	1.022 (0.967–1.080)	0.448
Smoking history	0.785 (0.362–1.704)	0.540	1.379 (0.576–3.303)	0.471	0.218 (0.029–1.673)	0.143
Atrial fibrillation	1.462 (0.450–4.744)	0.527	1.205 (0.284–5.117)	0.800	1.589 (0.208–12.155)	0.656
CAD	1.050 (0.253–4.356)	0.947	0.936 (0.126–6.942)	0.949	0.811 (0.106–6.204)	0.840
CKD	2.210 (0.928–5.264)	0.073	1.499 (0.565–3.975)	0.416	1.761 (0.230–13.471)	0.586
PCI performed	1.781 (0.746–4.250)	0.194	1.031 (0.386–2.754)	0.951	4.447 (0.581–34.035)	0.151
FBG	1.090 (0.962–1.235)	0.176	0.935 (0.790–1.107)	0.434	1.247 (0.745–2.088)	0.400
HbA1c	1.238 (1.041–1.472)	0.016	1.000 (0.763–1.311)	0.999	1.109 (0.597–2.060)	0.744
TC	0.800 (0.553–1.157)	0.236	0.772 (0.489–1.219)	0.267	0.821 (0.449–1.504)	0.524
LDL-C	0.739 (0.482–1.133)	0.166	0.711 (0.411–1.230)	0.223	0.764 (0.385–1.515)	0.440
Cr	1.008 (0.995–1.022)	0.219	1.011 (0.997–1.026)	0.132	0.994 (0.964–1.025)	0.694
eGFR	0.996 (0.982–1.011)	0.619	0.991 (0.975–1.007)	0.264	1.012 (0.986–1.039)	0.360
caIMR	1.027 (1.003–1.051)	0.028	1.015 (0.986–1.044)	0.310	1.023 (0.976–1.073)	0.334
Aspirin	0.463 (0.205–1.048)	0.065	0.606 (0.182–2.024)	0.416	0.272 (0.085–0.868)	0.028
P2Y12 receptor antagonist	0.874 (0.416–1.836)	0.722	0.371(0.156–0.883)	0.025	1.925 (0.431–8.609)	0.391
Statin	0.313 (0.123 − 0.800)	0.015	0.654 (0.196–2.181)	0.490	0.161 (0.036–0.722)	0.017
ACEI/ARB	1.280 (0.680 − 2.410)	0.445	1.058 (0.480–2.332)	0.889	1.300 (0.451–3.747)	0.627
Beta blocker	1.111 (0.590 − 2.093)	0.744	1.474 (0.655–3.317)	0.348	0.729 (0.254–2.088)	0.556
CCB	1.258 (0.674 − 2.347)	0.470	1.418 (0.655–3.072)	0.375	1.585 (0.531–4.734)	0.409

*DM* diabetes mellitus, *caIMR* coronary angiography-derived index of microcirculatory resistance, *BMI* body mass index, *PCI* percutaneous coronary intervention, *CKD* chronic kidney disease, *LVEF* left ventricular ejection fraction, *FBG* fasting blood glucose, *HbA1c* hemoglobin A1c, *TC* total cholesterol, *LDL-C* low-density lipoprotein-cholesterol, *Cr* creatinine, *eGFR* estimate glomerular filtration rate, *ACEI/ARB* angiotensin-converting-enzyme inhibitors/angiotensin receptor blockers, *CCB* calcium channel blocker, *CI* confidence interval, *HR* hazard ratio

**Table 5:** Multivariable analysis for clinical outcome

	All		DM		Non-DM	
	HR (95% CI)	P-value	HR (95% CI)	P-value	HR (95% CI)	P-value
caIMR ≥ 25	2.683 (1.343–5.361)	0.005	2.760 (1.066–7.146)	0.036	-*	
caIMR	-*		-*		-*	
CKD	-*		-*		-*	
Male	-*		-*		-*	
Hypertension	-*		-*		-*	
HbA1c	-*		-*		-*	
Statin	0.240 (0.088–0.649)	0.005	-*		0.163 (0.030–0.898)	0.037
Aspirin	-*		-*		0.208 (0.051–0.850)	0.029
P2Y12 receptor antagonist	-*		-*		-*	
LVEF	0.910 (0.876–0.945)	< 0.001	0.935 (0.894–0.978)	0.003	0.883 (0.814–0.958)	0.003

* variable with P-value > 0.05

*DM* diabetes mellitus; *caIMR* coronary angiography-derived index of microcirculatory resistance, *CKD* chronic kidney disease, *LVEF* left ventricular ejection fraction, *HbA1c* Hemoglobin A1c, *CI* confidence interval, *HR* hazard ratio

## Discussion

The present study is the first to evaluate the prognostic impact of CMD assessed by caIMR in DM patients with CCS. The main findings of this study were: (1) CMD assessed by caIMR was common among DM patients with CCS; (2) caIMR is an independent predictor associated with worsening clinical outcomes among DM patients with CCS. Our findings suggest that caIMR may facilitate an early and rapid measure of CMD in DM patients. Evaluating caIMR can provide a risk classification strategy for diabetic individuals.

Prior studies have shown that DM is a widespread independent risk factor for the development of cardiovascular disease, both DM and CCS are associated with an increased risk of cardiovascular morbidity and mortality [3–6]. It is increasingly recognized that CMD is an essential component of DM-associated CAD [1, 40–42]. Sara et al. among non-obstructive CAD patients, reported that CMD was found in 72.1% of diabetic patients, and CMD can result in either vasodilatory abnormality and/or abnormal vasoconstriction of the coronary microvessels [12]. CMD is found as an early feature of DM that may precede the development of the atherosclerotic disease of the epicardial arteries and contribute to the pathogenesis of myocardial ischemia [43, 44]. Clinical evidence demonstrated that CMD is a common finding among DM patients [45, 46] and may represent a potential determinant of adverse clinical outcomes [13–17]. A study by Cortigiani L et al. showed that in patients with type 2 diabetes, CMD prior to the involvement of the coronary artery was a significant and independent predictor of clinical outcomes [14]. Another study also indicated that coronary flow reserve (CFR), which reflects coronary microvascular function, is the most significant prognostic marker of composite outcome in DM patients but not in those without DM [16]. It has been also demonstrated that in the context of stable angina and nonobstructive CAD, prediabetes patients have a higher rate of coronary endothelial dysfunction than individuals with normoglycemia [47]. DM patients who had a reduced CFR demonstrated similar high mortality rates as those of non-diabetic patients with evidence of obstructive CAD [17]. Additionally, Gallinaro et al. evaluated the role of microvascular resistance reserve (MRR), a continuous thermodilution-derived novel index based on volumetric quantification of absolute flow and resistance, which is specific to the microvascular region and is operator-independent and used to quantify CMD; the authors demonstrated that CFR and MRR values among diabetics were significantly lower compared with nondiabetic patients [48].

The relationship between CMD and unfavorable clinical outcomes has been described in numerous past investigations using several diagnostic approaches, in which, wire-derived IMR was considered to be a more convenient and reliable tool for assessing the status of coronary microvasculature in the cardiac catheterization laboratory [25, 26]. IMR measured at the time of primary PCI in the patients with STEMI, NSTEMI, and stable CAD reliably predicts adverse events indicating the prognostic importance of CMD in these pathological states [27–30]. Moreover, previous evidence also shows that a high IMR in DM patients was associated with an increased risk of adverse events [15]. Despite the increasing evidence in favor of IMR studies, numerous factors such as longer procedural time, additional cost, technical complexity mainly related to pressure wire manipulation, and the need for adenosine infusion to achieve maximal hyperemia limit its use in routine clinical practice.

caIMR is a new simple angiography-based technique for assessing microvascular resistance, which is independent of epicardial coronary disease and specific to the microcirculation [31, 49]. While caIMR allows rapid and more cost-effective quantification of microvascular function, which can accurately predict wire-derived IMR [32, 33], measurement with caIMR has also been shown to predict future clinical outcomes in patients with STEMI and CAD [35, 50]. A study by Jordi et al. using angiography-derived IMR (NH-IMRangio) to assess CMD in Takotsubo Syndrome (TTS) patients found that NH-IMRangio values are associated with patterns of wall mobility abnormalities and the degree of ventricular dysfunction [51]. Additionally, our research team recently evaluated the prognostic value of caIMR in MINOCA patients and discovered that caIMR maintained good diagnostic performance and was a strong predictor of adverse risk among the MINOCA population [36]. Based on the published literature, caIMR ≥ 25 is used to define CMD, and this cut-off value is related to the poor prognosis of patients with CAD [31]. To the best of our knowledge, no previous studies have investigated the prognostic role of caIMR among DM patients with CCS. Our study demonstrated that patients with a high caIMR had a significantly higher MACE rate than patients with low caIMR. Besides, to investigate the prognostic role of caIMR and to compare the strength of its association with patient clinical outcomes, the patients were classified into DM and non-DM groups, and each of these groups was further classified into low and high caIMR groups. Among these groups, only DM patients with CMD (caIMR≥25) had a significantly higher risk of MACE than the other groups. The Kaplan-Meier survival curves also demonstrated an exceptionally high MACE risk in diabetic patients with high caIMR. Furthermore, after adjustment for critical covariates known to be associated with increased risk of MACE for diabetics showed that a caIMR ≥ 25 was still associated with increased risk of MACE among DM patients. As a result, high caIMR in DM patients is a powerful independent predictor of MACE. Although the exact mechanism driving CMD in DM is still unknown, in individuals with non-obstructive CAD, CMD is the result of either increased basal flow or decreased hyperemic flow or both. Accordingly, the association between high caIMR and DM patients perhaps is due to these unique pathophysiological mechanisms involved in DM patients as the latter have been demonstrated by a recent study [48]; however, this requires further elucidation. A previous study showed that, in patients with stable CAD, microvascular resistance assessed by IMR increases significantly after PCI, perhaps due to acute microvascular damage during the procedure, and dynamic changes in microvascular resistance are associated with PCI-related myocardial injury [52]. However, in the present study, the caIMR was measured after PCI in a majority of our patients; unfortunately, we do not have a pre-PCI caIMR values, so whether the caIMR increases after the PCI procedure is unclear. In addition, in our study, the myocardial injury was only found in 18 patients (7.9%) after PCI (see Additional file 1: Table S3); due to the small sample size of myocardial injury in this study, we did not find a correlation between post-PCI cTnT and caIMR; further prospective research studies with a large sample size are needed to explore the relationship of myocardial injury with the caIMR, and explore further the impact of these results on the long-term outcome.

Taken together, the main clinical implication of these findings is that caIMR can be used as an easy, quick, and cost-effective tool for microvascular function measurement in a catheter lab. In addition to DM patients, evaluating CMD by caIMR in patients with cardiovascular disease may enable physicians to identify the highest risk patients timely. Such patients may benefit most from closer follow-up and early intervention of novel therapies aimed at microvascular recovery. A recent study has demonstrated that glycemic control with anti-glycemic agents such as metformin is crucial for diabetics, and even little changes in glycemic level may reduce coronary endothelial dysfunction, subsequently reducing the high risk of cardiovascular events and improving clinical outcomes [47].

There are some limitations to our study. First, in our study majority of our patients underwent revascularization with PCI, and caIMR was measured post-PCI; therefore, any revascularization may potentially lead to transient alteration in the microvascular function resulting in a potential overestimation of the caIMR itself. Second, caIMR is a novel technique with minimal outcome data. Software of this nature is very operator-dependent and has a steep learning curve. Third, angiographic images were collected in a retrospective manner, which could influence the feasibility and reliability of caIMR analysis. Fourth, there is no specific cut-off point to define CMD in DM patients with CCS; however, we considered the widely spread cut-off point of 25 [31] to explain CMD among DM patients with CCS. Fifth, FBG was measured on the following day of hospital admission; unfortunately, our study lacks the serial plasma glucose data change during hospitalization and the follow-up period; therefore, we are unable to provide any details on whether FBG, acutely, would impact microcirculation; further studies are needed to confirm this association. Furthermore, despite observing an association between caIMR and clinical outcomes after adjusting for several potential confounders, the impact of unmeasured confounders cannot be ruled out. Additionally, this is a retrospective observational study; the sample size of this study population was relatively small with limited follow-up. Further large-scale studies are needed to validate the present results.

## Conclusion

This study demonstrates that CMD assessed by caIMR is common and is an independent predictor for MACE among diabetic patients with CCS. This finding may potentially enable the identification of high-risk patients who would benefit most from timely management with adjunctive therapeutic strategies.

## Electronic supplementary material

Below is the link to the electronic supplementary material.

Supplementary Material 1: Table S1: CAD characteristics of the study population. Table S2: Glucose-lowering therapy of DM patients. Table S3: Procedural characteristics

## Data Availability

The data analyzed in this study can be obtained from the corresponding author with a reasonable request.

## Abbreviations

CMD: coronary microvascular dysfunction
DM: diabetes mellitus
caIMR: coronary angiography-derived index of microcirculatory resistance
CCS: chronic coronary syndrome
MACE: major adverse cardiac events
IMR: the index of microcirculatory resistance
STEMI: st-elevated myocardial infarction
NSTEMI: non–ST-segment elevation myocardial infarction
CAD: coronary artery disease
MINOCA: myocardial infarction with non-obstructive coronary arteries
MI: myocardial infarction
CABG: coronary artery bypass graft surgery
FBG: fasting blood glucose
HbA1c: glycated hemoglobin A1c
TC: total cholesterol
LDL: low-density lipoprotein
PCI: percutaneous coronary intervention
BMI: body mass index
LVEF: left ventricular ejection fraction
CAG: coronary angiography
RCA: right coronary artery
LAD: left anterior descending artery
LCX: left circumflex artery
CFR: coronary flow reserve
NH-IMRangio: angiography-derived IMR
TTS: takotsubo Syndrome
