# Supplementary material for: Prognostic value of coronary microvascular dysfunction assessed by coronary angiography-derived index of microcirculatory resistance in diabetic patients with chronic coronary syndrome
Source: Cardiovasc Diabetol. 2022 Oct 29;21:222. doi: 10.1186/s12933-022-01653-y (PMC9618191; doi:10.1186/s12933-022-01653-y)
Supplement: Supplementary file 1 — Supplementary Material 1: Table S1: CAD characteristics of the study population. Table S2: Glucose-lowering therapy of DM patients. Table S3: Procedural characteristics [file 12933_2022_1653_MOESM1_ESM.docx]

**Table S1. CAD characteristics of the study population**

|  | **ALL** | | P-value | **DM** | | P-value | **Non-DM** | | P-value |
| --- | --- | --- | --- | --- | --- | --- | --- | --- | --- |
|  | caIMR≥25 (n=131) | caIMR<25 (n= 159) |  | caIMR≥25 (n= 59) | caIMR<25 (n= 43) |  | caIMR≥25 (n=72) | caIMR<25 (n= 116) |  |
| CAD, n (%) | 122(93.1) | 154(96.9) | 0.141 | 56(94.9) | 43(100.0) | 0.364 | 66(91.7) | 111(95.7) | 0.411 |
| 1- vessel disease | 54(41.2) | 61(38.4) | 0.621 | 23(39.0) | 17(39.5) | 0.955 | 31(43.1) | 44(37.9) | 0.485 |
| 2- vessel disease | 45(34.4) | 55(34.6) | 0.966 | 19(32.2) | 15(34.9) | 0.777 | 26(36.1) | 40(34.5) | 0.820 |
| 3- vessel disease | 23(17.6) | 38(23.9) | 0.187 | 14(23.7) | 11(25.6) | 0.830 | 9(12.5) | 27(23.3) | 0.068 |

*DM* diabetes mellitus, *caIMR* coronary angiography-derived index of microcirculatory resistance, *CAD* coronary artery disease.

**Table S2. Glucose-lowering therapy of DM patients**

|  | DM (n= 102) |
| --- | --- |
| Insulin, n (%) | 18 (17.6) |
| Oral glucose-lowering agents, n (%) | 76 (74.5) |
| Metformin, n (%) | 45 (44.1) |
| Glinides, n (%) | 17 (16.7) |
| Sulfonylureas, n (%) | 20 (19.6) |
| DPP-4 inhibitors, n (%) | 4 (3.9) |
| Thiazolidinediones, n (%) | 8 (7.8) |
| Alpha-glucosidase inhibitors, n (%) | 34 (33.3) |
| SGLT-2 inhibitors, n (%) | 3 (2.9) |

*DM* diabetes mellitus, *DPP-4* *inhibitors* dipeptidyl peptidase-4, *SGLT-2 inhibitors* sodium glucose cotransporter-2 inhibitors.

**Table S3. Procedural characteristics**

|  | All (n= 229) | DM (n= 86) | Non-DM (n=143) | P-value |
| --- | --- | --- | --- | --- |
| Percent stenosis (%) | 83.00±7.40 | 83.90±8.03 | 82.44±6.96 | 0.123 |
| Number of stents | 1.39±0.56 | 1.37±0.51 | 1.40±0.60 | 0.731 |
| Stent diameter (mm) | 2.90±0.48 | 2.92±0.44 | 2.89±0.50 | 0.613 |
| Stent length (mm) | 24.75±8.28 | 24.48±8.16 | 24.91±8.37 | 0.654 |
| caIMR | 26.46±11.05 | 29.54±12.48 | 24.61±9.68 | 0.002 |
| caFFR | 91.48±6.27 | 91.93±5.95 | 91.21±6.46 | 0.401 |
| cTnT (ng/ml) | 0.010±0.006 | 0.011±0.006 | 0.009±0.005 | 0.006 |
| Myocardial injury, n (%) | 18 (7.9) | 10 (11.6) | 8 (5.6) | 0.100 |
| Target vessel | 285 | 111 | 174 |  |
| LAD, n (%) | 152 (53.3) | 59 (53.2) | 93 (53.4) | 0.961 |
| LCX, n (%) | 62 (21.8) | 25 (22.5) | 37 (21.3) | 0.802 |
| RCA, n (%) | 71 (24.9) | 27 (24.3) | 44 (25.3) | 0.855 |

*DM* diabetes mellitus, *caFFR* coronary angiography-derived fractional flow reserve, *caIMR* coronary angiography-derived index of microcirculatory resistance, *cTnT* troponin T, *LAD* left anterior descending branch; *LCX* left circumflex coronary artery, *RCA* right coronary artery.
